# Effect of acute exercise and hypoxia on markers of systemic and mucosal immunity

**DOI:** 10.1007/s00421-016-3380-4

**Published:** 2016-04-29

**Authors:** Ida S. Svendsen, Erlend Hem, Michael Gleeson

**Affiliations:** School of Sport, Exercise and Health Sciences, Loughborough University, Loughborough, LE11 3TU UK; Norwegian Olympic and Paralympic Committee and Confederation of Sports, Oslo, Norway

**Keywords:** Altitude, Endurance, Cortisol, S-IgA, Cytokine, Lymphocytes

## Abstract

**Purpose:**

To determine how immune markers are affected by acute hypoxic exercise at the same relative intensity.

**Methods:**

Twelve endurance-trained males (age: 28 ± 4 years, $\dot{V}$O_2max_: 63.7 ± 5.3 mL/kg/min) cycled for 75 min at 70 % of altitude-specific $\dot{V}$O_2max_, once in normoxia (N) and once in hypobaric hypoxia equivalent to 2000 m above sea-level (H). Blood and saliva samples were collected pre-, post- and 2 h post-exercise.

**Results:**

Participants cycled at 10.5 % lower power output in H vs. N, with no significant differences in heart rate (*P* = 0.10) or rating of perceived exertion (*P* = 0.21). Post-exercise plasma cortisol was higher in H vs. N [683 (95 % CI 576–810) nmol/l vs. 549 (469–643) nmol/l, *P* = 0.017]. The exercise-induced decrease in CD4:CD8 ratio was greater in H vs. N (−0.5 ± 0.2 vs. −0.3 ± 0.2, *P* = 0.019). There were no significant between-trial differences for adrenocorticotropic hormone, plasma cytokines, antigen-stimulated cytokine production, salivary immunoglobulin-A or lactoferrin. However, there was a main trial effect for concentration [*F*(11) = 5.99, *P* < 0.032] and secretion [*F*(11) = 5.01, *P* < 0.047] of salivary lysozyme, with this being higher in N at every time-point.

**Conclusion:**

Whether the observed differences between H and N are of sufficient magnitude to clinically impair host defence is questionable, particularly as they are transient in nature and since other immune markers are unaffected. As such, acute hypoxic exercise likely does not pose a meaningful additional threat to immune function compared to exercise at sea level, provided that absolute workload is reduced in hypoxia so that relative exercise intensity is the same.

## Introduction

Various forms of hypoxic training are used by competitive endurance athletes, both to acclimatise before competition at altitude (Fulco et al. 2000) and to improve exercise performance at sea level (Levine and Stray-Gundersen 1997; Stray-Gundersen et al. 2001). Exercise in hypoxic conditions typically causes greater physiological stress than exercise at sea level (Bailey and Davies 1997). Taking into account this augmented stress response to exercise, coupled with reports of increased resting levels of adrenaline, cortisol and interleukin-6 at high altitude (Mazzeo et al. 2001), it seems reasonable to hypothesise that hypoxic training might pose a greater threat to immune function compared to equivalent training at sea level. Indeed, both anecdotal evidence from athletes and coaches, and scientific literature point towards an increased incidence of opportunistic infectious illness during and immediately after altitude training (Bailey et al. 1998; Gore et al. 1998). This may be due, at least in part, to an impairment of T cell-mediated immunity during altitude exposure (Meehan et al. 1988; Facco et al. 2005; Oliver et al. 2013; Pyne et al. 2000). Meanwhile, other aspects of immunity do not appear to respond negatively to hypoxic training. Tiollier et al. (2005) found that salivary secretory immunoglobulin-A (S-IgA) decreased progressively in elite cross-country skiers training at 1200 m above sea-level (masl). However, this decrease was not exacerbated in athletes sleeping at 2500–3500 masl compared to the control group who slept at 1200 masl, and Born et al. (2015) actually observed increases in both salivary S-IgA and alpha-amylase following intermittent hypoxic training. Natural killer cell activity (Klokker et al. 1995; Wang and Wu 2009) and B-cell function (Pyne et al. 2000) appear to be unaffected or increased by hypoxic training, as does neutrophil function (Wang and Chiu 2009; Chouker et al. 2005). Nevertheless, it is still unclear how many aspects of immune function respond to hypoxic exercise, and particularly to the types of exercise and levels of hypoxia that athletes are typically exposed to during training or competition at altitude. Furthermore, it is well known that maximal oxygen uptake ($\dot{V}$O_2max_) is reduced at altitude, with the magnitude of this reduction being proportional to the level of hypoxia (Ferretti et al. 1997). Some previous studies that have investigated differences in immune responses to an acute bout of exercise performed in normoxic and hypoxic conditions have matched trials based on absolute workload, such as a specific running speed or power output (Klokker et al. 1995; Mazzeo et al. 2001). However, because of the reduction in $\dot{V}$O_2max_, the same absolute workload performed in hypoxia will result in higher relative exercise intensity (% $\dot{V}$O_2max_). To determine whether the differences in responses observed are due to hypoxia *per-se*, rather than this increase in relative exercise intensity, it is useful to instead match trials based on a fraction of altitude-specific $\dot{V}$O_2max_. This is also of greater relevance to endurance athlete populations, since they will typically train at a similar, or even a lower, relative exercise intensity at altitude as when they are at sea level (Gore et al. 1998).

Traditional forms of hypoxic training involve prolonged sojourns at natural altitude. Today, however, an increasing number of athletes utilise “live low, train high” protocols, during which only a proportion of training sessions are performed in acute hypoxia. Although the efficacy of this type of hypoxic training in improving sea-level performance remains somewhat equivocal (McLean et al. 2014) it has become popular due to being more cost-effective and convenient than traveling to natural altitude. Nevertheless, it is not yet understood how many aspects of the immune system respond to this type of hypoxic training. The aim of the current study was therefore to determine how markers of systemic and mucosal immunity are affected by an acute bout of endurance exercise in hypobaric hypoxia equivalent to 2000 masl, compared to exercise at the same relative intensity in normoxia.

## Methods

### Participants

Twelve endurance-trained males volunteered their written, informed consent to take part in the study [mean (± SD) age: 28 ± 4 years, body mass 79.1 ± 5.9 kg, $\dot{V}$O_2max_ 63.7 ± 5.3 mL/kg/min, 5.06 ± 0.45 L/min], which was approved by the regional ethics committee of southern Norway. Before the start of the study, participants completed a health-screening questionnaire. Participants could be included if they were between 18 and 40 years of age, participating in regular endurance training, currently healthy and without URS or use of any medication during the past 4 weeks. Exclusion criteria were smoking, suffering from, or with a history of, cardiac, hepatic, pulmonary, renal, neurological, haematological, psychiatric, or gastrointestinal illness, or exposure to natural or simulated altitude (equivalent to ≥1000 m above sea level) during the past 8 weeks.

### Laboratory visits

On the first laboratory visit, 1–2 weeks before the first experimental trial, participants completed a continuous, incremental exercise test to volitional exhaustion in normobaric conditions on an electromagnetically braked cycle ergometer (Lode Excalibur Sport, Groningen, The Netherlands) for determination of $\dot{V}$_2max_. The test began at 60 W with increments of 35 W every 3 min. Rating of perceived exertion (RPE) was noted during the final minute of each stage and heart rate measured continuously using short-range telemetry (Polar, Kempele, Finland). Expired gas was collected and analysed continuously using a computerized metabolic system with mixing chamber (Oxycon Pro, Erich Jaeger GmbH, Hoechberg, Germany) for determination of minute ventilation, oxygen uptake and carbon dioxide production. The gas analysers were calibrated with certified calibration gases of known concentrations before every test. The flow turbine was calibrated before every test with a 3 L calibration syringe (CareFusion, Hoechberg, Germany). The work rate corresponding to 70 % $\dot{V}$O_2max_ was then calculated from the $\dot{V}$O_2_-work rate relationship using a linear equation. The same relative workload for the hypoxic trial was calculated based on data from a previous study conducted in the decompression chamber showing that, for individuals of this training status, $\dot{V}$O_2max_ decreases by approximately 10.4 % from 59.8 ± 7.4 to 53.6 ± 6.7 ml/kg/min in hypobaric hypoxia equivalent to 2000 masl (Hårklau 2011), a finding supported by earlier studies that have similarly observed a reduction of ~10 % (Eriksen 2002; Sylta 2009). As such, the following formula was used to predict $\dot{V}$O_2max_ at 2000 masl:

$${\text{Predicted }}\dot{V} {\text{O}}_{2\hbox{max} } {\text{ at }}2000{\text{ masl}} = 0.896* \dot{V} {\text{O}}_{2\hbox{max} } {\text{ at sea level}}$$

The same linear regression equation used to calculate workload in normoxia was then applied to determine the workload corresponding to 70 % of the predicted $\dot{V}$O_2max_ at 2000 masl.

For both main experimental trials, participants arrived at the laboratory at 07:45 am following an overnight fast of at least 10 h, and having abstained from caffeine for a minimum of 12 h. Participants were also required to abstain from alcohol and strenuous exercise for at least 24 h before each trial and, in an effort to standardize nutritional status, asked to record and replicate their dietary intake the day before. For both trials, participants completed a standardised 10 min warm-up at 55 % of altitude specific $\dot{V}$O_2max,_ followed by 75 min at 70 % altitude specific $\dot{V}$O_2max_, on an electromagnetically braked cycle ergometer (Lode Excalibur Sport, Groningen, Netherlands) inside a decompression chamber (Norsk undervannsteknikk A/S, Haugesund, Norway). Following the exercise bout, participants remained inside the decompression chamber for a 2 h resting recovery. During the normoxic trial (N), the decompression chamber was switched off, and the doors remained open. For the hypoxic trial (H), the pressure was adjusted to 800 mbar (equivalent to 2000 masl at 17 °C). Oxygen and carbon dioxide levels inside the chamber were continuously monitored throughout the trials. Carbon dioxide levels were maintained via three CO_2_ scrubbers containing soda lime, and oxygen was supplied continuously at a rate equal to oxygen consumption to preserve normal ambient composition (~20.9 % O_2_, ~0.04 % CO_2_). To confirm that the same relative exercise was achieved in both trials, heart rate was measured continuously via short range telemetry (RCX3, Polar, Kempele, Finland), RPE was recorded every 10 min, and blood lactate concentration was measured from a finger prick blood sample using a portable blood lactate analyser (Lactate Pro LT-1710, Arkray, Kyoto, Japan) every 20 min during exercise.

### Saliva collection

Saliva samples were collected at rest immediately before entry into the decompression chamber (PRE), immediately after cessation of exercise (POST), and after a 2 h recovery period (2 h POST). After sitting quietly for a few minutes, participants were instructed to swallow to empty the mouth before collecting unstimulated whole saliva into a pre-weighed 7 mL screw-top vial over a predetermined time period (3–4 min). During the collection time, participants were instructed to remain seated, leaning forwards with their head tilted down, allowing the saliva to dribble passively into the collection tube with minimal orofacial movement. Saliva volume was determined by weighing to the nearest milligram, with saliva density estimated to be 1.0 g/ml (Cole and Eastoe 2014). Saliva flow rate was then calculated by dividing the volume by the collection time. Samples were centrifuged for 3 min at 14,000*g* before being stored at −80 °C until analysis.

### Blood sampling

Blood samples were collected at the same time points as saliva. On each occasion, a venous blood sample (11 mL) was obtained by venepuncture from an antecubital vein. Blood was collected into two vacutainer tubes (Becton–Dickinson, Oxford, UK) containing either lithium heparin or K_3_EDTA as anticoagulant.

### Plasma cytokines, adrenocorticotropic hormone (ACTH) and cortisol

The K_3_EDTA sample was centrifuged for 10 min at 1500*g* and 4 °C. Plasma was aliquoted and stored at −20 °C until analysis. Plasma concentrations of interferon gamma (IFN-γ), tumor necrosis factor alpha (TNF-α), interleukin- (IL-)1α, IL-6, IL-8, IL-10, epidermal growth factor (EGF), vascular endothelial growth factor (VEGF) and monocyte chemoattractant protein-1 (MCP-1) were determined with an Evidence Investigator System using the cytokine biochip array EV 3623 (Randox, County Antrim, UK). The intra-assay coefficient of variation was <5 % for all measured cytokines. Plasma concentrations of cortisol were determined using a commercially available solid phase competitive enzyme-linked immunosorbent assay (IBL International, Hamburg, Germany), with analytical sensitivity of 2.46 ng mL^−1^ and intra-assay coefficient of variation of <3.0 %. Plasma concentrations of ACTH were determined using a commercially available two-site enzyme-linked immunosorbent assay (Biomerica, California), with analytical sensitivity of 0.22 pg ml^−1^ and intra-assay coefficient of variation of <6.0 %. Plates were read on a microtitre plate reader at 450 nm. All samples were assayed in duplicate.

### Antigen-stimulated cytokine production

Stimulated whole blood culture production of IFN-γ, TNF-α, IL-1α, IL-1β, IL-2, IL-4, and IL-10 was determined as follows: for each sample, 0.25 mL heparinized whole blood was added to 0.75 mL RPMI medium (Sigma Chemicals, Poole, UK) with added stimulant at a dilution of 1:2000. The dilution used was based on a separate experiment (unpublished data), which established the dose–response curve for the measured cytokines over the dilution range of 1:200–1:20,000. The stimulant used was a commercially available multiantigen (DTaP/IPV/Hib) vaccine containing diphtheria, tetanus, acellular pertussis, poliomyelitis, and Haemophilus influenza type b antigens (Pediacel vaccine, Sanofi Pasteur, Maidenhead, UK). All participants in the current study had received the DTaP/IPV/Hib vaccine as infants. The whole blood culture was then incubated for 24 h at 37 °C and 5 % CO_2_. Following centrifugation for 4 min at 13,000*g* in a microcentrifuge, supernatants were collected and stored frozen at −20 °C until analysis. Cytokine concentrations were determined with an Evidence Investigator System using the cytokine biochip array EV 3623 (Randox, County Antrim, UK). The intra-assay coefficient of variation was <5 % for all measured cytokines.

*Salivary antimicrobial proteins* Commercially available ELISA kits were used to determine salivary concentrations of S-IgA (Salimetrics, Philadelphia, USA) lysozyme (Biomedical Technologies, USA) and lactoferrin (Calbiochem, Merck KGaA, Darmstadt, Germany). For Lysozyme and Lactoferrin, saliva was diluted 500× before analysis. Secretion rates for each of the salivary antimicrobial proteins were calculated by multiplying saliva flow rate by concentration. All samples were assayed in duplicate. The intra-assay CV for S-IgA, lysozyme and lactoferrin were 1.8, 5.3 and 3.0 %, respectively.

### Lymphocyte subsets

For a subset of 8 participants, fluorescent-conjugated monoclonal antibodies were used to identify specific cell surface markers (CD3, CD8, CD4, CD25, CD127) via four colour flow-cytometry with True Volumetric Absolute Counting (Partec CyFlow ML, Germany) and FloMax analysis software (Quantum Analysis GmbH). Briefly, either 10 μL of human regulatory T cell cocktail (Becton–Dickinson Biosciences, Oxford, UK) or 10 μL of FITC anti-human CD3 + 10 μL PeCy7 CD4 + 10 μL PE CD8 (Becton–Dickinson Biosciences) were added to 120 μL heparinized whole blood and incubated in the dark for 20 min on ice. Erythrocytes were then lysed by adding 1.5 mL of lyse solution (FACS lysis buffer, Becton–Dickinson Biosciences). The sample was subsequently incubated for a further 10 min, before centrifugation at 1500*g* for 6 min. The supernatant was aspirated and the cells re-suspended in phosphate buffered saline solution containing 0.1 % bovine serum albumin and 2 mM EDTA. The mixture was centrifuged for a further 6 min at 1500*g*, the supernatant aspirated and the cells re-suspended in 1000 μL phosphate buffered saline. Forward-scatter versus side-scatter plots were used to gate lymphocytes based on size and density, with 50,000 lymphocyte events acquired per analysis. CD4+ lymphocytes were further gated to identify CD25+ cells, and those that were also CD127low/-. This was based on the finding that CD127 expression inversely correlates with FoxP3 (Liu et al. 2006) and can therefore be used to identify T regulatory cells. CD3+ cells were further gated to identify cells that were CD4+ (T helper) and CD8+ (T suppressor). CV for determination of all lymphocyte subsets was <6 %.

### Statistical analyses

A priori analyses were conducted using G*Power 3 (Faul et al. 2007). Based on these data a sample size of 12 was predicted to be sufficient to detect statistically significant differences between immune responses in normoxia and hypoxia, and minimize the type 2 error rate. Data are presented as mean ± standard deviation (SD) unless otherwise stated. All statistical analyses were performed using IBM SPSS Statistics 22.0. The Shapiro–Wilk test was used to determine whether data were normally distributed. A two-way repeated measures analysis of variance was used to determine whether there were significant differences between hypoxic and normoxic trials PRE, POST and 2 h POST. A post hoc test with Bonferroni correction for multiple comparisons was used to determine the location of variance. Variables found to be significantly non-normal were log transformed before analysis (Box and Cox 1964), and are presented as back-transformed geometric mean with 95 % confidence interval (CI). Based on the results of Mauchly’s sphericity test, Greenhouse-Geisser corrections were applied for epsilon <0.75 and Huynd-Feldt corrections applied for epsilon >0.75 where violations of sphericity were identified. Statistical significance was accepted at the *P* < 0.05 level.

## Results

**Fig. 1:**
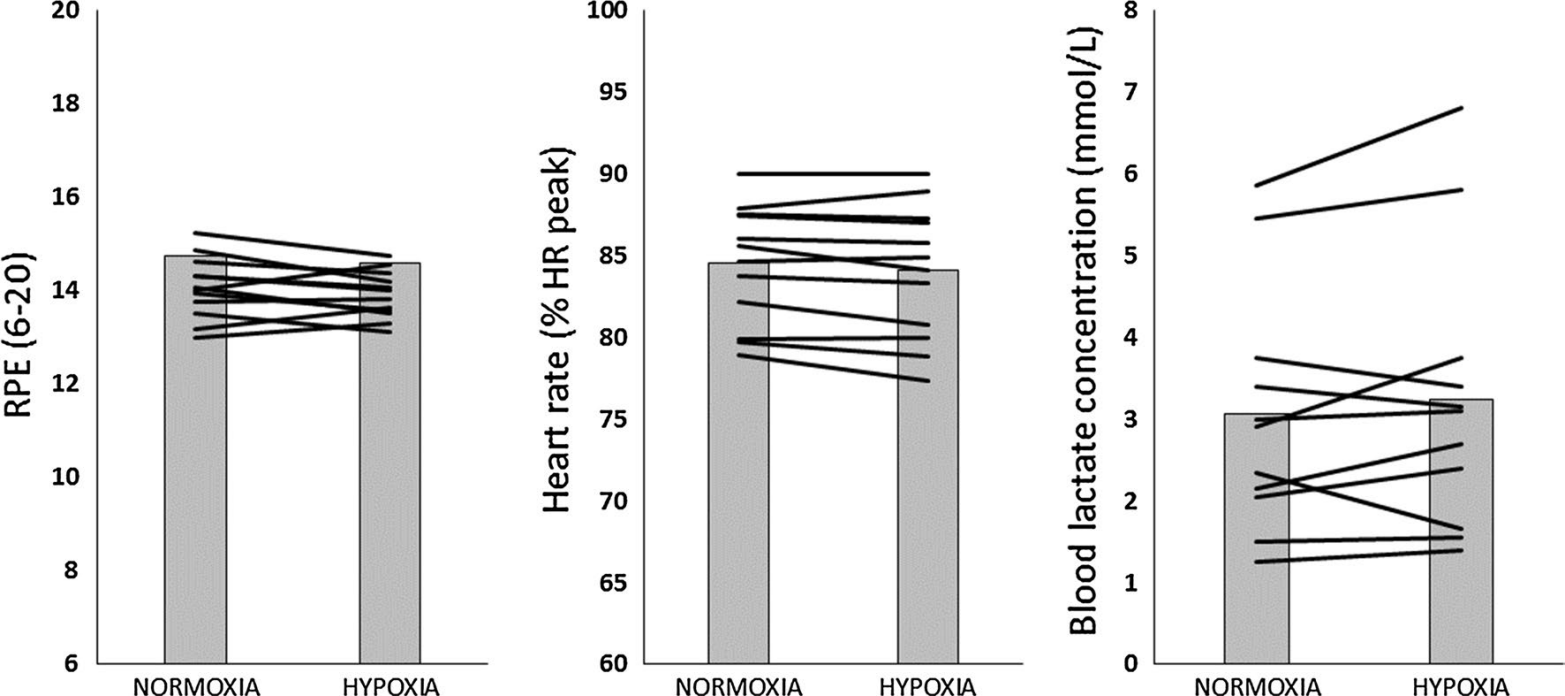
Average RPE, heart rate and blood lactate concentration during exercise in normoxia and hypoxia. *Bars* represent group mean, *lines* represent individual responses

On average, participants cycled at 10.5 % lower power output in H compared to N (191 ± 26 vs. 213 ± 30 W). There were no significant differences in heart rate, blood lactate concentration or RPE between trials (Fig. 1).

### Plasma cytokines, ACTH and cortisol

**Fig. 2:**
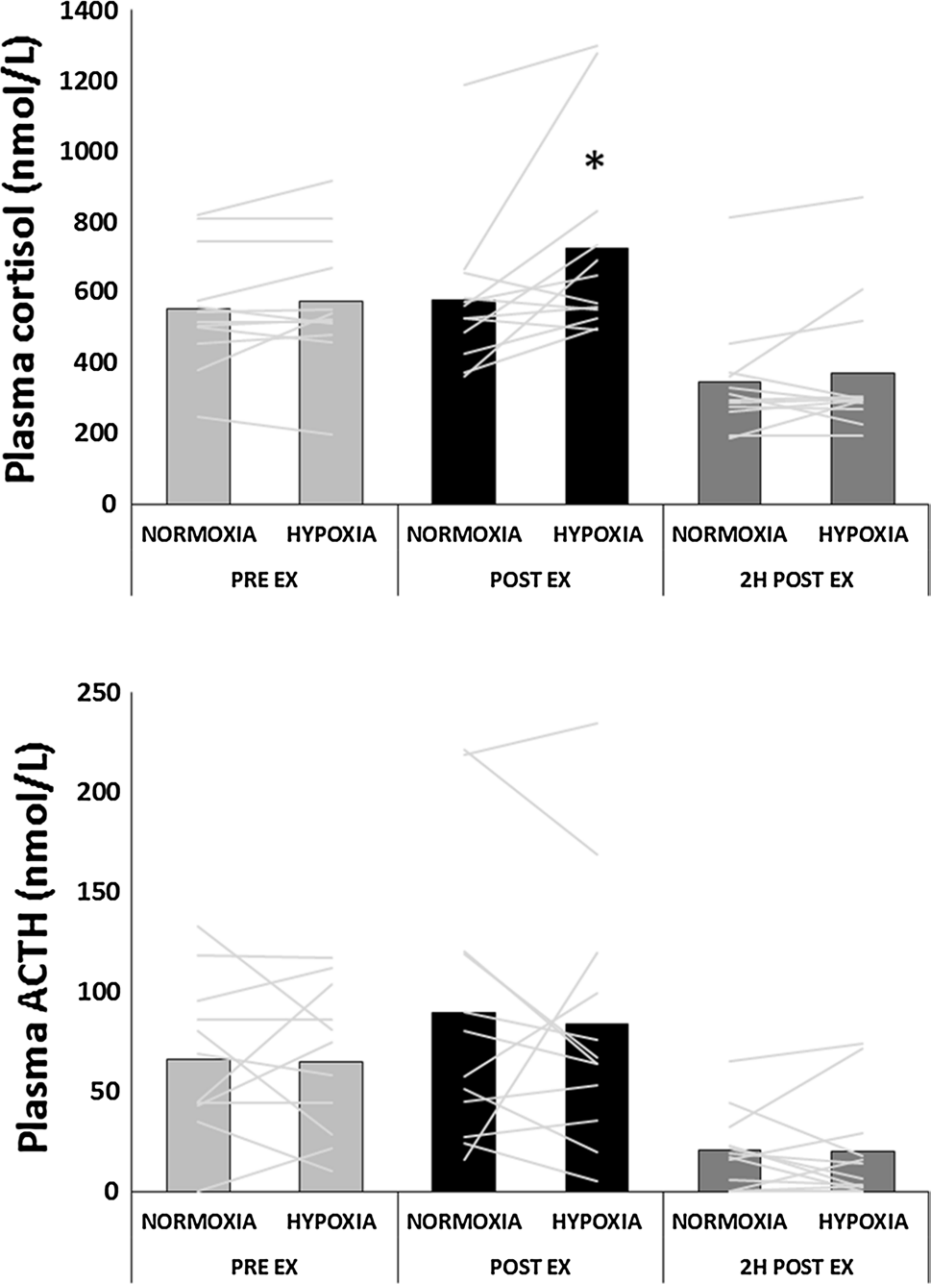
Plasma cortisol and ACTH before, immediately after, and 2 h after cycling exercise in normoxia and hypoxia. *Bars* represent group mean, *lines* represent individual responses. *Significant difference from normoxia (*P* < 0.05). ^#^Both trials are significantly different from pre-exercise and post-exercise (*P* < 0.01)

In both trials, plasma cortisol and ACTH (Fig. 2) concentrations were significantly lower 2 h post exercise compared to pre-exercise and immediately post-exercise. Plasma cortisol immediately post-exercise was significantly higher in H compared to N 683 (95 % CI 576–810) nmol/l vs. 549 (469–643) nmol/l, *P* = 0.017). There were no significant differences between trials at any time point for plasma ACTH. Table 1 shows plasma cytokine concentrations before, immediately after and 2 h after exercise in normoxic and hypoxic conditions. There were no significant differences between trials at any time point for any of the measured cytokines. However, plasma IL-6, IL-8 and MCP1 concentrations were significantly elevated post and 2 h post-exercise in both H and N, with only IL-6 remaining elevated at 2 h post-exercise. Plasma VEGF was significantly reduced immediately post-exercise in both conditions, before returning to baseline 2 h post exercise.

**Table 1:** Plasma cytokine concentrations before, immediately after and 2 h after exercise in normoxia and hypobaric hypoxia

	PRE	POST	2 h POST
IL-1a (pg/ml)			
Normoxia	0.30 (0.17–0.42)	0.25 (0.15–0.36)	0.26 (0.17–0.36)
Hypoxia	0.29 (0.21–0.37)	0.31 (0.22–0.41)	0.37 (0.17–0.36)
IL-6 (pg/ml)			
Normoxia	0.70 (0.58–0.85)	2.71 (2.11–3.49)^#^	1.10 (0.85–1.44)^#,†^
Hypoxia	0.70 (0.55–0.88)	2.53 (2.00–3.20)^#^	1.09 (0.83–1.41)^#,†^
IL-8 (pg/ml)			
Normoxia	2.26 (1.90–2.62)	3.47 (2.90–4.04)^#^	2.71 (2.10–3.33)^†^
Hypoxia	2.19 (1.84–2.55)	3.31 (2.53–4.09)^#^	2.71 (2.01–3.40)^†^
IL-10 (pg/ml)			
Normoxia	1.10 (0.84–1.44)	1.50 (1.10–2.11)	1.10 (0.89–1.35)^†^
Hypoxia	0.69 (0.39–1.16)	1.55 (1.12–2.21)	1.10 (0.82–1.49)^†^
TNFα (pg/ml)			
Normoxia	2.44 (1.99–2.88)	2.44 (2.09–2.80)	2.24 (1.86–2.62)
Hypoxia	2.18 (1.88–2.48)	2.27 (1.99–2.55)	2.48 (1.98–2.48)
EGF (pg/ml)			
Normoxia	11.3 (8.9–14.0)	17.4 (12.1–23.8)	17.9 (13.5–22.9)
Hypoxia	12.7 (8.9–17.2)	16.3 (10.1–23.9)	11.6 (7.2–16.9)
VEGF **(**pg/ml)			
Normoxia	14.7 (12.2–17.8)	11.5 (8.8–15.0)^#^	14.9 (11.5–19.3)^†^
Hypoxia	13.3 (10.3–17.3)	12.0 (9.0–16.0)^#^	14.1 (11.4–17.4)^†^
MCP1 (pg/ml)			
Normoxia	107 (93–120)	124 (111–137)^#^	105 (93–117)^†^
Hypoxia	103 (92–113)	121 (108–135)^#^	111 (94–127)^†^

Values are mean (95 % CI)

^#^Significantly different from pre-exercise (*P* < 0.01)

^†^Significantly different from post-exercise (*P* < 0.01)

### Antigen-stimulated cytokine production

**Fig. 3:**
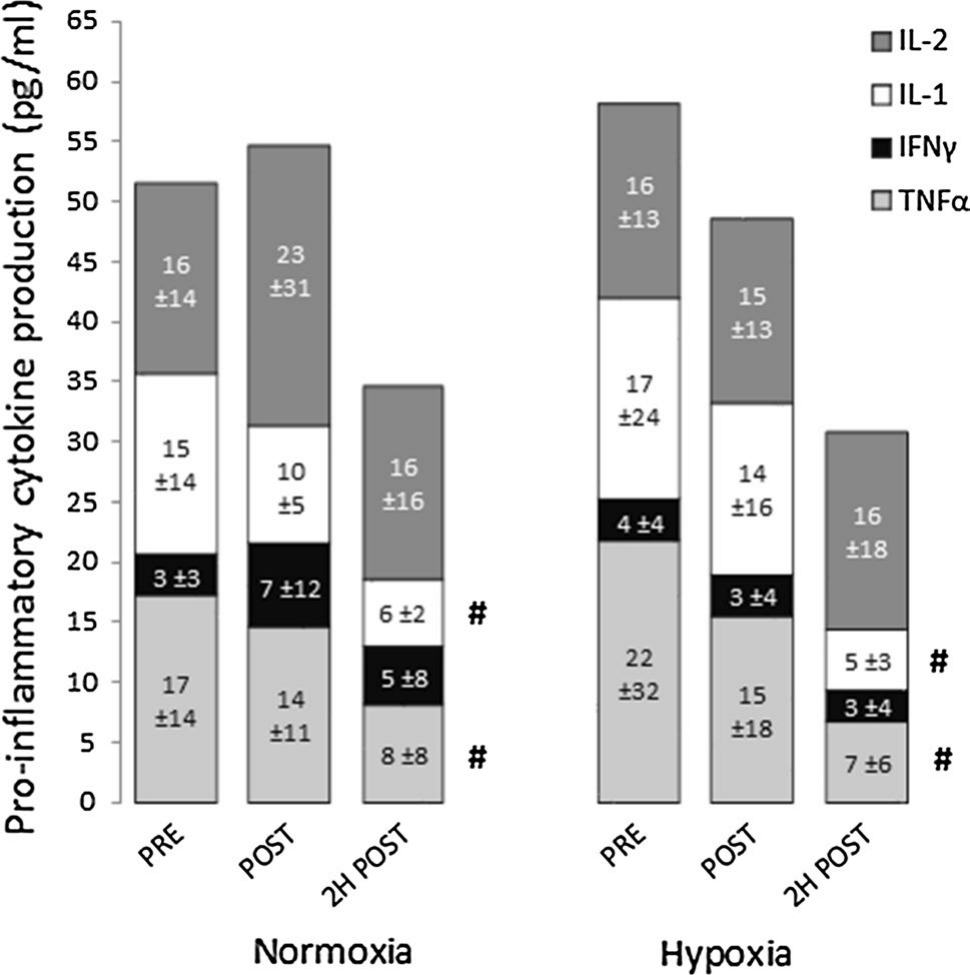
Pro-inflammatory cytokine production by whole blood culture in response to a multi-antigen challenge. *IL-2* interleukin 2, *IL-1* interleukin 1 (α + β), *IFNγ* interferon gamma, *TNFα* tumour necrosis factor alpha. Data are mean ± SD. ^#^Significant main effect of time for TNFα and IL-1 (*P* < 0.05)

**Fig. 4:**
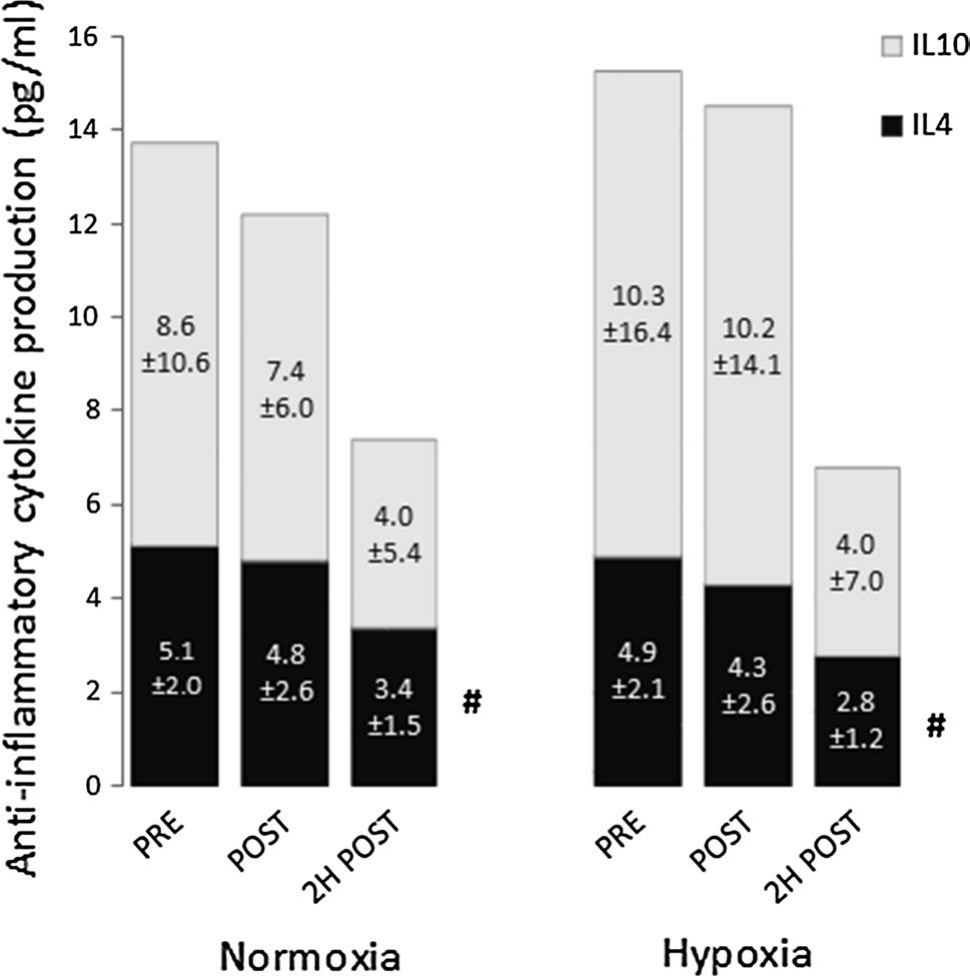
Anti-inflammatory cytokine production by whole blood culture in response to a multi-antigen challenge. *IL-10* interleukin 10, *IL-4* interleukin 4. Data are mean ± SD. ^#^Significant main effect of time for IL-4 (*P* < 0.01)

Figures 3 and 4 show in vivo pro-inflammatory and anti-inflammatory cytokine production in whole blood culture in response to a multi-antigen challenge. Production of TNFα, IL-1 and IL-4 were significantly reduced 2 h post-exercise. There were no significant differences between the two trials.

### Salivary antimicrobial proteins

There were no significant differences in S-IgA concentration or secretion rate (Table 2). Both concentration and secretion rates of salivary lysozyme (*P* = 0.005 and *P* = 0.014, respectively) and lactoferrin (*P* = 0.0009 and *P* = 0.002, respectively) were significantly increased post-exercise, with lactoferrin remaining significantly elevated 2 h post-exercise. There was a significant main effect of hypoxia for both concentration [*F*(11) = 5.99, *P* = 0.032] and secretion rate [*F*(11) = 5.01, *P* = 0.047] of salivary lysozyme, with this being higher in N at every time-point.

**Table 2:**
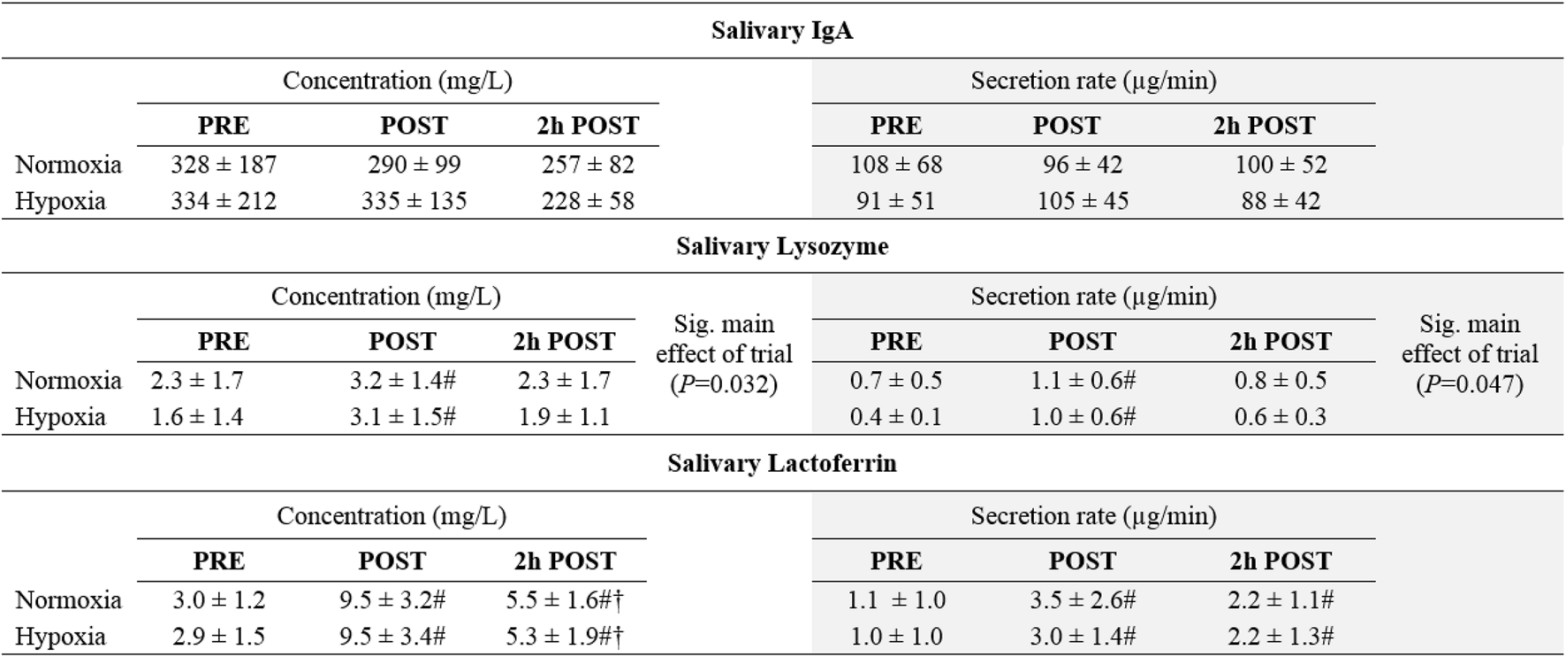
Concentrations and secretion rates of salivary antimicrobial proteins before, immediately after and 2 h after exercise in normoxia and hypobaric hypoxia

Data are mean ± SD

^#^Significantly different from pre-exercise (*P* < 0.01)

^†^Significantly different from post-exercise (*P* < 0.01)

### Lymphocyte subsets

**Fig. 5:**
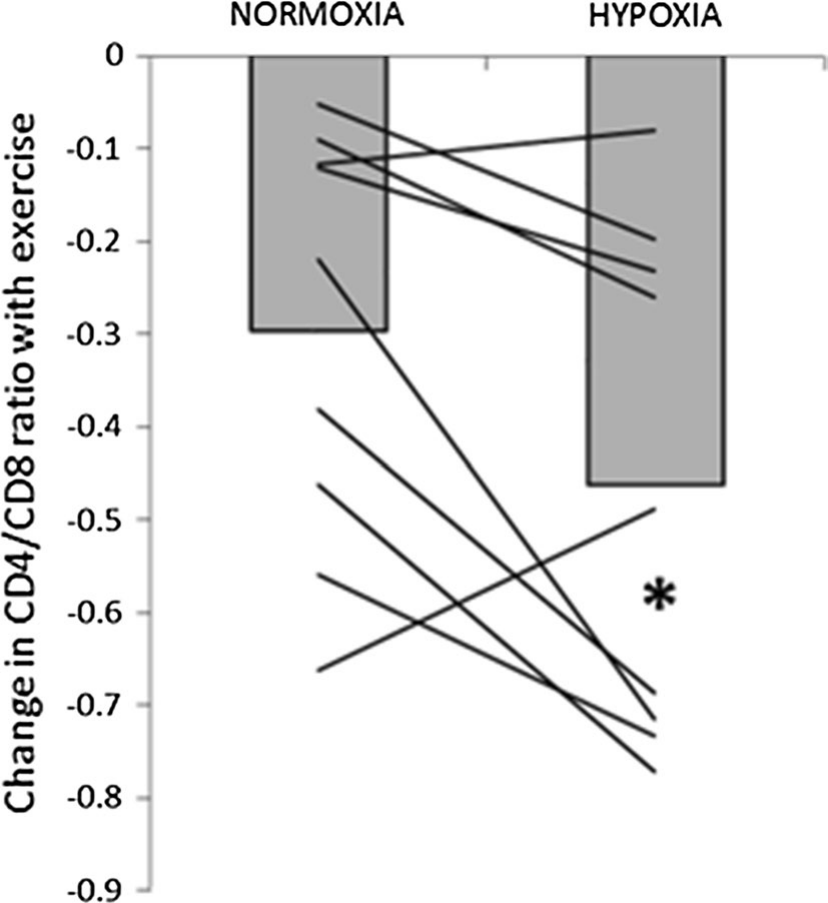
Exercise-induced change in CD4/CD8 ratio. *Bars* represent group mean, *lines* represent individual changes. *Significant difference from normoxia (*P* < 0.05)

Total number of circulating lymphocytes (*P* = 0.018), T cells (CD3+; *P* = 0.008), T helper cells (CD3+CD4+; *P* = 0.025) and T suppressor cells (CD3+CD8+; *P* = 0.002) were all significantly reduced 2 h post- exercise (Table 3). The relative number of lymphocytes as a percentage of total leukocytes was also significantly reduced post (*P* = 0.0013) and 2 h post-exercise in both trials (*P* = 0.0001). There was no change in the absolute or relative number of T regulatory cells. The exercise-induced decrease in CD4:CD8 ratio was significantly greater in H compared to N (−0.5 ± 0.2 vs. −0.3 ± 0.2, *P* = 0.019), ES(d) = 0.6, Fig. 5), but in both trials this returned to baseline by 2 h post-exercise. There were no significant differences between conditions for any of the other variables.

**Table 3:** Lymphocyte subsets before, immediately after and 2 h after exercise in normoxia (N) and hypobaric hypoxia (H)

		Absolute (×10^9^ cells/L)			Relative^a^ (% total leukocytes/lymphocytes)		
		PRE	POST	2 h POST	PRE	POST	2 h POST
Lymphocytes	N	1.54 ± 0.53	1.59 ± 0.51	1.30 ± 0.46^**†**^	32 ± 9	23 ± 6^**#**^	15 ± 7^**#,†**^
	H	1.49 ± 0.47	1.75 ± 0.35	1.02 ± 0.27^**†**^	32 ± 9	23 ± 6^**#**^	12 ± 7^**#,†**^
T (*CD3*+)	N	0.79 ± 0.28	0.74 ± 0.24	0.53 ± 0.16^**†**^	52 ± 10	48 ± 13	43 ± 15
	H	0.75 ± 0.31	0.81 ± 0.31	0.52 ± 0.12^**†**^	50 ± 13	46 ± 12	52 ± 14
T helper (*CD3*+*CD4*+)	N	0.46 ± 0.12	0.39 ± 0.10	0.32 ± 0.10^**#,†**^	30 ± 5	26 ± 6	27 ± 11
	H	0.43 ± 0.15	0.39 ± 0.11	0.31 ± 0.06^**#,†**^	29 ± 8	23 ± 7	32 ± 9
T suppressor (*CD3*+*CD8*+)	N	0.32 ± 0.18	0.32 ± 0.14	0.20 ± 0.08^**†**^	20 ± 8	20 ± 8	16 ± 4
	H	0.30 ± 0.18	0.38 ± 0.22	0.20 ± 0.08^**†**^	19 ± 8	21 ± 9	19 ± 7
T regulatory (*CD4*+*CD25*+*CD127*−)	N	0.03 ± 0.02	0.03 ± 0.02	0.02 ± 0.01	2.0 ± 0.6	1.7 ± 0.7	1.2 ± 0.5
	H	0.03 ± 0.01	0.03 ± 0.01	0.02 ± 0.01	2.0 ± 0.5	1.6 ± 0.8	1.8 ± 1.1

Data are mean ± SD

^#^Significantly different from pre-exercise (*P* < 0.05)

^†^Significantly different from post-exercise (*P* < 0.05). *n* = 8

^a^Relative numbers of lymphocytes are presented as percentage of total leukocytes, while lymphocyte subsets are presented as percentage of total lymphocytes

## Discussion

The main finding of the current study is that, despite a somewhat augmented stress response as indicated by higher post-exercise plasma cortisol, an acute exercise bout in hypobaric hypoxia equivalent to 2000 masl does not appear to pose any meaningful additional threat to immune function compared to exercising at the same relative intensity in normoxia. However, previous studies have found that hypoxic exercise at the same absolute exercise intensity can exacerbate immune disturbances, highlighting the importance of accurately controlling exercise intensity at altitude. Lundy and Steensberg (2004) found that there was no difference in post-exercise plasma IL-6 concentration with either acute or chronic hypoxic exposure when participants cycled at the same relative exercise intensity, whilst IL-6 release was augmented when exercising at the same absolute intensity. This is in agreement with the current study where there was no effect of hypoxia on post-exercise plasma concentrations of IL-6, or any of the other measured cytokines, when cycling at 70 % of altitude specific $\dot{V}$O_2max_. The 134 nmol/L difference in post-exercise cortisol was not of sufficient magnitude to influence cytokine production and is unlikely to meaningfully impair host defence, particularly since this difference was no longer present following 2 h recovery.

It is well established that exercise causes changes in circulating lymphocytes subsets, including a transient decrease in CD4:CD8 ratio (Fry et al. 1992; Nieman et al. 1991). The results of the current study suggest that this exercise-induced decrease is more pronounced when exercising in hypobaric hypoxia, even when relative exercise intensity is the same. This was principally driven by a greater increase in circulating CD8+ cells in H, but also, although to a lesser extent, by an exacerbated post-exercise reduction in circulating CD4+ cells. The normal resting range for healthy individuals is 1.5–2.5, although this can vary considerably with age and genetics (Amadori et al. 1995). In four participants, exercise in hypoxia was associated with a post-exercise CD4:CD8 ratio of <1.0. Although the clinical relevance of this is not clear, CD4:CD8 ratio is an important marker of immune health. HIV (Fauci 1993) as well as a number of acute viral infections such as Influenza, cytomegalovirus and Epstein-Barr virus are typically associated with an inversion of the CD4:CD8 ratio (Gratama et al. 1986; Amadori et al. 1995). Although plasma catecholamine concentrations were not measured in the current study, it is possible to speculate that the greater reduction in CD4:CD8 ratio observed in the hypoxic trial may have been due to higher levels of circulating adrenaline. Compared to CD4+ cells, CD8+ lymphocytes have more β-adrenergic receptors (Landmann et al. 1984; Van Tits et al. 1990) which likely explains the greater mobilization of CD8+ cells observed with exercise. Although only speculation, it has been previously shown that the catecholamine response in trained individuals is augmented when exercising in hypoxia at the same absolute intensity (Kjaer et al. 1988), and to a lesser degree also at the same relative intensity (Kjaer et al. 1988). This is supported by the higher post-exercise cortisol observed in the hypoxic trial in the current study, since the magnitude of the cortisol response typically reflects the magnitude of the catecholamine response. It is worth noting that there was no longer a significant difference in either plasma cortisol or CD4:CD8 ratio between trials 2 h post-exercise. As such, any negative effects of acute hypoxic exercise on host defence appear to be short-lived. Nevertheless, athletes utilising this training may benefit from taking some additional precautions to minimise pathogens exposure during the 2 h window immediately post-hypoxic exercise.

The observed reductions in TNF-α and IL-1 release in response to antigen challenge 2 h post-exercise in both trials correspond with previous studies reporting a temporal suppression of mitogen- or LPS-stimulated production of type 1 cytokines following prolonged exercise (Baum et al. 1997; Drenth et al. 1995; Lewicki et al. 1988; Starkie et al. 2000). Conversely, while IL-4 production was also significantly reduced, previous studies have found that acute endurance exercise has little or no effect on IL-4 release in response to mitogen stimulation (Lancaster et al. 2004, 2005). However, in the current study these changes likely primarily reflect changes in the number of responder cells in the culture (i.e., changes in circulating leukocyte subsets). This is a weakness of the method used, since it does not allow identification of which cells in the culture are producing specific cytokines. On the other hand, using viral and/or bacterial antigens to stimulate cytokine production in whole blood culture probably most closely represents the natural environment, avoiding artefacts from cell isolation and preparation and enabling normal interactions between antigens and immune components within the natural hormonal milieu. Furthermore, it gives a measure of the total capacity to respond to an immune challenge at that specific time-point. The results of the current study indicate that the post-exercise reduction in cytokine production is not exacerbated when exercise of the same relative intensity is performed in hypobaric hypoxia.

Tiollier et al. (2005) found no influence of living high and training low on salivary S-IgA. Pilardeau et al. (1990), meanwhile, found that saliva flow rate was significantly increased when exercising at 4350 masl compared to at sea level, while alpha-amylase concentration was unaffected. However, we are not aware of any previous studies that have investigated the effects of acute exercise and hypoxia on salivary S-IgA, lysozyme and lactoferrin, which are postulated to play an important role in mucosal immunity and first-line defence against invading microbials (Dürr et al. 2006; Fahlman and Engels 2005; Neville et al. 2008; Tenovuo 1989). Exercise-induced changes in salivary S-IgA and lactoferrin do not appear to be affected by moderate hypoxia when relative exercise intensity is the same. Lysozyme is a major antimicrobial protein in saliva and, as such, is an important component of the innate immune system. It displays a wide range of antimicrobial activities, principally via cleaving of glycosidic bonds between the C-1 of N-acetylmuramic acid and the C-4 of N-acetylglucosamine in the peptidoglycan of the bacterial cell wall (Jollès and Jollès 1984). As such, a reduction in salivary lysozyme concentration/secretion has the potential to compromise host defence against bacterial pathogens. However, the significant trial effect observed for salivary lysozyme in our study can likely be largely explained by lower lysozyme concentration and secretion rate already at baseline in the hypoxic trial, rather than an effect of hypoxia. It is possible to speculate that the difference in salivary lysozyme concentration and secretion rate between H and N pre-exercise may have been due to an anticipatory effect, since participants were not blind to the condition. Walsh et al. (1999) found that salivary α-amylase secretion was reduced immediately pre-exercise, and the authors speculated that this may have been due to anticipatory psychological stress. We are not aware of any studies that have examined whether such an anticipatory effect is present for salivary lysozyme. However, Yang and colleagues (2002) have reported a negative correlation between self-perceived work stress and salivary lysozyme secretion rate. Unfortunately, it was not possible to blind participants in the present study, since we were not able to reduce the barometric pressure inside the decompression chamber without the participant noticing. Overall, the results of the current study do not indicate that cycling for 75 min at 70 % $\dot{V}$O_2max_ negatively influences mucosal immunity, regardless of whether exercise is performed in normoxia or hypobaric hypoxia. Indeed, both concentration and secretion rates of lysozyme and lactoferrin were increased post-exercise in both conditions. This corresponds with previous studies that have reported similarly elevated concentrations of salivary lysozyme and lactoferrin following intense endurance exercise at sea level (Allgrove et al. 2008; West et al. 2010).

It should be noted that these results are not representative of the effects of a prolonged altitude sojourn, during which an athlete is continuously exposed to hypoxia, typically for several weeks. However, the exercise stimulus and hypoxic dose used in the current study are of high relevance to the increasing number of athletes who use “Live low, train high” protocols (McLean et al. 2014), since this type of hypoxic training involves performing a proportion of training sessions in acute hypoxia, while otherwise residing at sea level.

## Conclusion

Acute hypoxic exercise is associated with somewhat higher plasma cortisol and lower CD4:CD8 ratio immediately post-exercise. However, whether these differences are of sufficient magnitude to meaningfully impair host defence is questionable, particularly since they appear transient in nature and since the other measured markers of immunity were unaffected. As such, athletes can likely implement “Live low, train high” regimes without notably increasing their risk of infection, although a little additional care to minimise pathogen exposure during the 2 h window immediately post-hypoxic exercise may be pertinent. Furthermore, since previous studies have shown that hypoxic exercise at the same absolute workload can significantly exacerbate immune disturbances, this highlights the importance of accurately adjusting and monitoring exercise intensity during hypoxic training.
